# CYP19A1 polymorphisms associated with coronary artery disease and circulating sex hormone levels in a Chinese population

**DOI:** 10.18632/oncotarget.21626

**Published:** 2017-10-07

**Authors:** Yajie Meng, Dilare Adi, Yun Wu, Yongtao Wang, Mayila Abudoukelimu, Ding Huang, Xiang Ma, Cheng Liu, Ting Wang, Fen Liu, Bangdang Chen, Mintao Gai, Xiaocui Chen, Zhenyan Fu, Yitong Ma

**Affiliations:** ^1^ Department of Cardiology, First Affiliated Hospital of Xinjiang Medical University, Urumqi, P.R. China; ^2^ Xinjiang Key Laboratory of Cardiovascular Disease Research, Urumqi, P.R. China

**Keywords:** CYP19A1, coronary artery disease, sex hormone, polymorphisms

## Abstract

**Background:**

The relationship between CYP19A1 genetic polymorphisms and coronary artery disease (CAD) remains unclear. Thus, the aim of the present study was to investigate the association of CYP19A1 genetic polymorphisms with CAD in Han and Uygur populations and to characterize the association between the levels of sex hormones and aromatase with single-nucleotide polymorphisms (SNPs) in CYP19A1 genes in Chinese women.

**Results:**

There were significant differences in the genotype distributions of rs2236722 and rs4646 between CAD patients and control subjects in the Uygur population. The rs4646 was found to be associated with CAD in the dominant model (CC vs. CA + AA) and the additive model (CA vs. CC + AA) (both *P* ≤ 0.001). The difference remained statistically significant after multivariate adjustment (OR = 0.483, 95% CI: 0.338–0.690, *P* = 0.000; and OR = 1.844, 95% CI: 1.300–2.617, *P* = 0.001, respectively). In normal Uygur postmenopausal women, there were significant differences in the genotype distributions of rs4646 and the circulating hormone and aromatase levels between CAD patients and control subjects. The differences in estradiol and aromatase levels remained statistically significant after multivariate adjustment (OR = 0.889, 95% CI: 0.817–0.969, *P* = 0.007; and OR = 0.947, 95% CI: 0.936–0.957, *P* = 0.000, respectively). Additionally, there were differences in sex hormone levels between the different ethnicities among the Xinjiang Chinese population.

**Materials and Methods:**

Among a total of 1,064 Han individuals (614 men and 450 women) and 790 Uygur individuals (484 men and 306 women), 498 postmenopausal women (265 Han and 233 Uygur individuals) were selected. Four SNPs (rs2236722, rs2304463, rs4646, and rs4275794) were genotyped using the improved multiplex ligation detection reaction (iMLDR) technique. The estradiol and testosterone levels were determined using a radioimmunoassay based on GC-2016γ. In addition, an enzyme-linked immunosorbent assay (ELISA) was performed to determine the serum P450 aromatase levels.

**Conclusions:**

The results of this study indicate that the rs2236722 and rs4646 of the CYP19A1 gene are associated with CAD and circulating sex hormone levels in the Xinjiang population of China.

## INTRODUCTION

Coronary artery disease (CAD) is a complex multifactorial cardiovascular disease that is influenced by both genetics and environmental risk factors. CAD is considered a major cause of mortality worldwide, and the incidence of coronary events in women is increasing, with most events occurring after the onset of menopause [1–2]. Previous studies have suggested that estrogen reduction is an important factor in the increased incidence of coronary heart disease among postmenopausal women [3]. There is also evidence that sex hormones influence the risk of developing CAD, suggesting that declining levels of endogenous estrogen with age, the higher prevalence of obesity, and altered body fat distribution result in a pro-atherogenic metabolic environment and the subsequent development of significant vascular endothelial dysfunction [4–5].

**Figure 1:**
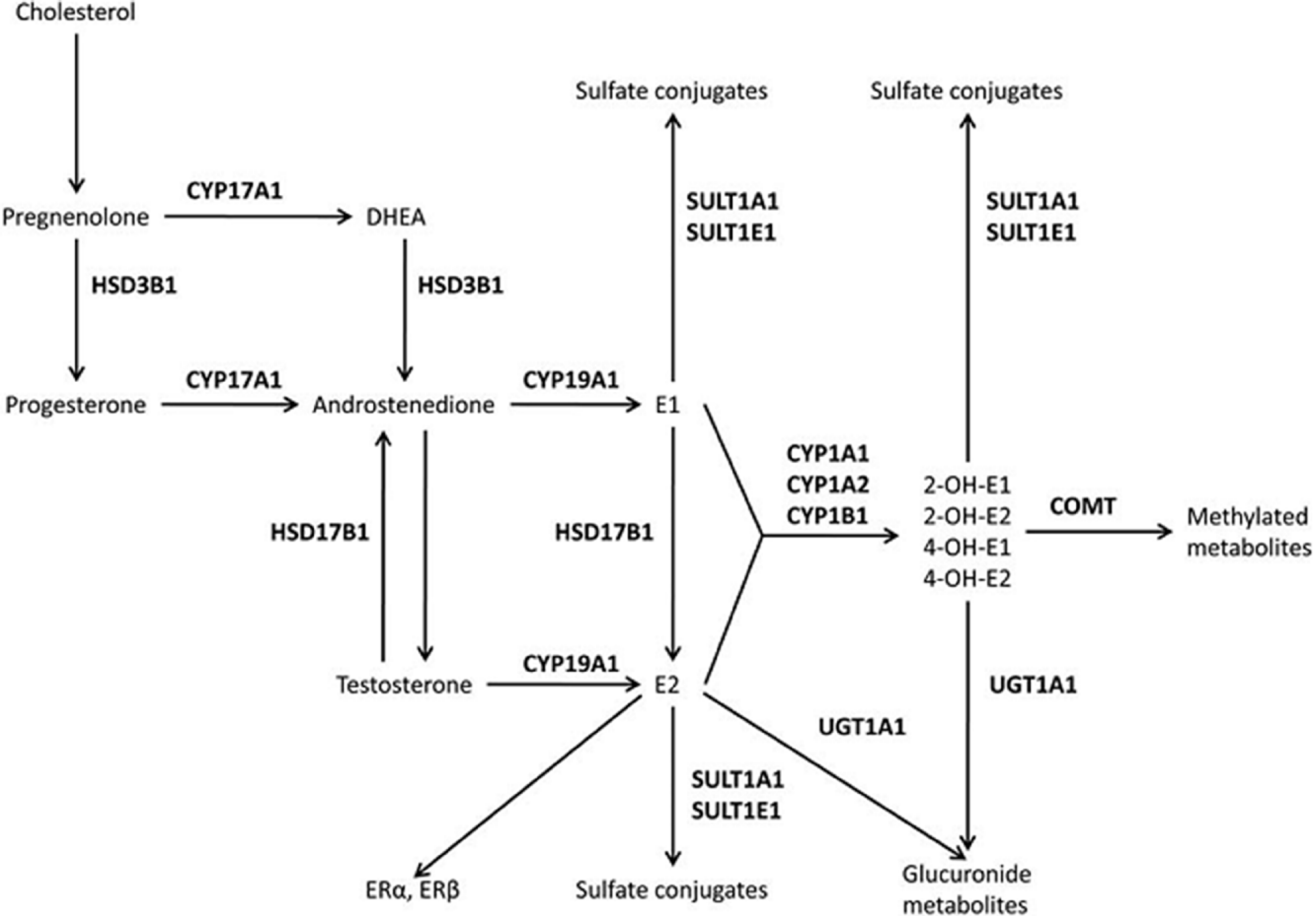
The estrogen pathway Hydroxysteroid dehydrogenases (HSD3B1 and HSD17B1), cytochrome P450 (CYP1A1, CYP1A2, CYP17A1, CYP19A1, and CYP1B1), catechol-O-methyltransferase (COMT), uridine diphospho-glucuronosyltransferase (UGT1A1), sulfotransferases (SULT1A1 and SULT1E1), and estrogen receptors (ER) alpha and beta.

In the estrogen pathway, the cytochrome P450 aromatase gene (CYP19A1) encodes a key enzyme involved in the conversion of androstenedione and testosterone to estrone (E1) and 17β-estradiol (E2), respectively (Figure 1). Many studies have examined whether genetic variations in CYP19A1 are associated with cardiovascular diseases [6–8]. However, despite these efforts, the potential molecular mechanisms linking estrogen-related genes with CAD risk remain incompletely understood.

Herein, we present a case-control study designed to assess the association between the levels of sex hormones (total testosterone and estradiol) and P450 aromatase levels based on single-nucleotide polymorphisms (SNPs) in the CYP19A1 gene to investigate the relationship between CYP19A1 and CAD in the Chinese population. Moreover, we analyzed the correlations among aromatase, total testosterone, and estradiol.

## RESULTS

### Characteristics of the study participants and frequencies of the SNPs

The present study evaluated two ethnic groups (1,046 Han and 790 Uygur subjects). The clinical and metabolic characteristics of the study populations are shown separately for Han and Uygur participants in Tables 1 and 2.

**Table 1:** Characteristics of the subjects (Han Chinese population)

	Total			Men			Women		
	CAD	Controls	*P*	CAD	Controls	*P*	CAD	Controls	*P*
Number (*n*)	611	453		421	193		190	260	
Age, mean (SD)	59.65 ± 11.12	59.0 ± 9.55	0.134	59.10 ± 11.71	59.0 ± 9.3	0.284	60.32 ± 8.24	58.22 ± 9.71	0.169
EH (%)	336 (55)	180 (39.7)	0.000	223 (53)	80 (41.5)	0.008	113 (59.5)	100 (38.5)	0.000
Diabetes (%)	155 (25.4)	46 (10.2)	0.000	105 (24.9)	35 (18.1)	0.062	50 (26.3)	11 (4.2)	0.000
Smoking (%)	282 (46.2)	134 (29.6)	0.000	277 (68.8)	127 (65.8)	0.999	5 (2.6)	7 (2.7)	0.968
Drinking (%)	309 (50.6)	105 (23.2)	0.000	254 (60.3)	96 (49.7)	0.014	55 (28.9)	9 (3.5)	0.000
BMI, mean (SD)	25.85 ± 3.35	24.49 ± 3.29	0.000	26.1 ± 3.39	25.48 ± 3.04	0.026	25.28 ± 3.29	23.77 ± 3.29	0.000
Glu (mmol/L)	6.36 ± 2.16	5.51 ± 1.89	0.000	6.31 ± 2.7	6.05 ± 2.49	0.257	6.45 ± 3.5	5.11 ± 1.30	0.000
TG (mmol/L)	2.11 ± 1.62	1.75 ± 1.48	0.000	2.20 ± 1.78	2.25 ± 1.85	0.753	1.92 ± 1.21	1.37 ± 0.98	0.000
TC (mmol/L)	4.65 ± 1.11	4.38 ± 2.87	0.037	4.23 ± 1.30	4.86 ± 1.18	0.000	4.69 ± 2.54	4.48 ± 1.02	0.230
HDL (mmol/L)	1.12 ± 0.84	1.28 ± 0.55	0.010	1.06 ± 0.83	1.25 ± 0.54	0.001	1.25 ± 0.84	1.31 ± 0.56	0.466
LDL (mmol/L)	3.06 ± 1.33	2.58 ± 1.11	0.000	3.04 ± 1.31	2.46 ± 1.03	0.000	3.07 ± 1.34	2.82 ± 1.24	0.049
UA (µmol/L)	325.54 ± 147.62	290.90 ± 83.95	0.000	347.08 ± 161.55	331.17 ± 85.48	0.199	277.8 ± 94.97	261.01 ± 69.12	0.031
Cr (µmol/L)	77.51 ± 35.36	76.48 ± 22.72	0.580	82.82 ± 38.44	90.98 ± 19.49	0.006	65.73 ± 23.46	65.72 ± 18.62	0.996
BUN (mmol/L)	6.26 ± 7.59	4.83 ± 1.42	0.000	6.44 ± 7.92	5.17 ± 1.46	0.002	5.86 ± 6.79	4.58 ± 1.33	0.003

Continuous variables are expressed as the mean ± SD. Categorical variables are expressed as percentages.

BMI, body mass index; BUN, blood urea nitrogen; Cr, creatinine; DM, diabetes mellitus; Glu, glucose; TG, triglyceride; TC, total cholesterol;

HDL, high-density lipoprotein; LDL, low-density lipoprotein; and UA, uric acid.

The *P*-value of the continuous variables was calculated using the independent-sample *t*-test. The *P*-value of the categorical variables was calculated by Fisher’s exact test.

**Table 2:** Characteristics of subjects (Uygur Chinese population)

	Total			Men			Women		
	CAD	Controls	*P*	CAD	Controls	*P*	CAD	Controls	*P*
Number (*n*)	403	387		316	168		87	219	
Age, mean (SD)	55.66 ± 9.85	52.85 ± 8.55	0.342	54.87 ± 8.09	52.26 ± 8.16	0.581	59 ± 8.17	53.31 ± 8.8	0.867
EH (%)	217 (53.8)	172 (44.4)	0.008	164 (51.90)	75 (44.60)	0.129	53 (60.9)	97 (44.3)	0.090
Diabetes (%)	90 (23.3)	31 (8.1)	0.000	64 (21)	14 (8.5)	0.001	26 (31.7)	17 (7.8)	0.000
Smoking (%)	174 (43.2)	79 (20.5)	0.000	173 (54.7)	75 (45.2)	0.046	1 (1.1)	4 (1.8)	0.673
Drinking (%)	173 (42.9)	34 (8.8)	0.000	147 (46.5)	34 (20.2)	0.000	26 (29.9)	0 (0)	0.000
BMI, mean (SD)	27.11 ± 3.78	26.36 ± 4.45	0.011	27.04 ± 3.68	26.33 ± 4.21	0.540	27.35 ± 4.12	26.37 ± 4.64	0.089
Glu (mmol/L)	6.08 ± 2.49	5.12 ± 1.89	0.000	5.88 ± 2.15	5.11 ± 1.70	0.000	6.83 ± 3.37	5.11 ± 2.02	0.000
TG (mmol/L)	2.04 ± 1.34	1.63 ± 1.11	0.000	1.96 ± 1.30	1.63 ± 1.09	0.070	2.34 ± 1.42	1.67 ± 1.13	0.000
TC (mmol/L)	4.67 ± 3.01	4.43 ± 1.09	0.147	4.56 ± 2.48	4.30 ± 1.08	0.213	5.08 ± 4.40	4.53 ± 1.09	0.034
HDL (mmol/L)	0.99 ± 0.9	1.21 ± 0.45	0.000	0.94 ± 0.83	1.24 ± 0.46	0.000	1.11 ± 1.09	1.19 ± 0.44	0.534
LDL (mmol/L)	2.72 ± 1.40	2.67 ± 1.41	0.656	2.69 ± 1.45	2.73 ± 1.42	0.727	2.86 ± 1.18	2.63 ± 1.42	0.169
UA (µmol/L)	313.86 ± 99.99	252.38 ± 79.78	0.000	323.81 ± 92.36	278.96 ± 81.80	0.000	276.8 ± 117.79	232.16 ± 72.07	0.002
Cr (µmol/L)	81.70 ± 37.29	72.26 ± 28.34	0.000	83.30 ± 34.93	80.42 ± 32.40	0.383	75.58 ± 44.92	66.06 ± 23.02	0.075
BUN (mmol/L)	6.14 ± 7.19	5.20 ± 1.50	0.013	5.9 ± 5.75	5.38 ± 1.52	0.248	7.02 ± 1.09	5.06 ± 1.47	0.122

Continuous variables are expressed as the mean ± SD. Categorical variables are expressed as percentages.

BMI, body mass index; BUN, blood urea nitrogen; Cr, creatinine; DM, diabetes mellitus; Glu, glucose; TG, triglyceride; TC, total cholesterol;

HDL, high-density lipoprotein; LDL, lo-density lipoprotein; and UA, uric acid.

The *P*-value of the continuous variables was calculated by the independent-sample *t*-test. The *P*-value of the categorical variables was calculated by Fisher’s exact test.

Tables 3 and 4 show the distribution of the CYP19A1 genotypes among the Han and Uygur populations. The genotype and allele distributions among the Han and Uygur patients and control participants for each SNP were consistent with the predicted Hardy-Weinberg equilibrium values (data not shown).

**Table 3:** Genotype and allele distributions among Han patients with CAD and control participants

		Total			Men			Women		
		CAD *n* (%)	Controls *n* (%)	*P*	CAD *n* (%)	Controls *n* (%)	*P*	CAD *n* (%)	Controls *n* (%)	*P*
SNP1 rs2236722 genotype	AA	592 (96.9)	434 (95.8)	0.379	400 (96)	189 (97.9)	0.422	188 (98.9)	245 (94.2)	0.01
	GA	18 (2.9)	19 (4.2)		16 (3.8)	4 (2.1)		2 (1.1)	15 (5.8)	
	GG	1 (0.2)	0 (0)		1 (0.2)	0 (0)		0 (0)	0 (0)	
										
Dominant model	AA	592 (96.9)	434 (95.8)	0.346	400 (96)	189 (97.9)	0.213			
	GA + GG	19 (3.1)	19 (4.2)		17 (4)	4 (2.1)				
Recessive model	GG	1 (0.2)	0 (0)	0.389	1 (0.2)	0 (0)	0.498			
	AA + GA	610 (99.8)	453 (100)		420 (99.8)	193 (100)				
Additive model	GA	18 (2.9)	19 (4.2)	0.272	16 (3.8)	4 (2.1)	0.263			
	AA + GG	593 (97.1)	434 (95.8)		405 (96.2)	189 (97.9)				
										
SNP2 rs2304463 genotype	CC	175 (28.6)	123 (27.2)	0.408	132 (31.4)	60 (31.1)	0.998	43 (22.6)	63 (24.2)	0.124
	CA	334 (54.7)	240 (53)		215 (51.1)	99 (51.3)		119 (62.9)	141 (54.2)	
	AA	102 (16.1)	90 (19.9)		74 (17.6)	34 (17.6)		28 (14.7)	56 (21.5)	
										
Dominant model	CC	175 (28.6)	123 (27.2)	0.593	132 (31.4)	60 (31.1)	0.947	43 (22.6)	63 (24.2)	0.693
	CA + AA	436 (71.4)	330 (72.8)		289 (68.6)	133 (68.9)		147 (77.4)	197 (75.8)	
Recessive model	AA	102 (16.1)	90 (19.9)	0.183	74 (17.6)	34 (17.6)	0.991	28 (14.7)	56 (21.5)	0.067
	CC + CA	509 (83.3)	363 (80.1)		347 (82.4)	159 (82.4)		162 (85.3)	204 (78.5)	
Additive model	CA	334 (54.7)	240 (53)	0.586	215 (51.1)	99 (51.3)	0.958	119 (62.9)	141 (54.2)	0.075
	CC + AA	277 (45.3)	213 (47.0)		99 (51.3)	94 (48.7)		71 (37.4)	119 (45.8)	
										
SNP3 rs4646 genotype	CC	354 (57.9)	243 (53.6)	0.377	232 (55.1)	100 (51.8)	0.608	122 (64.2)	143 (55)	0.143
	CA	212 (34.7)	173 (38.2)		157 (37.3)	80 (41.5)		55 (28.9)	93 (35.8)	
	AA	45 (7.4)	37 (8.2)		32 (7.6)	13 (6.7)		13 (6.8)	74 (9.2)	
										
Dominant model	CC	354 (57.9)	243 (53.6)	0.163	232 (55.1)	100 (51.8)	0.447	122 (64.2)	143 (55)	0.05
	CA + AA	257 (42.1)	210 (46.4)		189 (44.9)	93 (48.2)		68 (35.8)	117 (45)	
Recessive model	AA	45 (7.4)	37 (8.2)	0.627	32 (7.6)	13 (6.7)	0.703	13 (6.8)	74 (9.2)	0.362
	CC + CA	566 (92.6)	416 (91.8)		389 (92.4)	180 (93.3)		177 (93.2)	236 (90.8)	
Additive model	CA	212 (34.7)	173 (38.2)	0.241	157 (37.3)	80 (41.5)	0.326	55 (28.9)	93 (35.8)	0.128
	CC + AA	399 (65.3)	280 (61.87)		264 (62.7)	113 (58.5)		135 (71.7)	167 (64.2)	
										
SNP4 rs4275794 genotype	TT	415 (67.9)	307 (67.8)	0.975	279 (66.3)	129 (66.8)	0.963	136 (71.6)	178 (68.5)	0.63
	CT	179 (29.3)	132 (29.1)		128 (30.4)	57 (29.5)		51 (26.8)	75 (28.8)	
	CC	17 (2.8)	14 (3.1)		14 (3.3)	7 (3.6)		3 (1.6)	7 (2.7)	
										
Dominant model	TT	415 (67.9)	307 (67.8)	0.985	279 (66.3)	129 (66.8)	0.89	136 (71.6)	178 (68.5)	0.477
	CT + CC	196 (32.1)	146 (32.2)		142 (33.7)	64 (33.2)		54 (28.4)	82 (31.5)	
Recessive model	CC	17 (2.8)	14 (3.1)	0.775	14 (3.3)	7 (3.6)	0.849	3 (1.6)	7 (2.7)	0.429
	TT + CT	594 (97.2)	439 (96.9)		407 (96.7)	186 (96.4)		187 (98.4)	253 (97.3)	
Additive model	CT	179 (29.3)	132 (29.1)	0.956	128 (30.4)	57 (29.5)	0.827	51 (26.8)	75 (28.8)	0.64
	TT + CC	432 (70.7)	321 (70.9)		293 (69.6)	136 (70.5)		139 (73.2)	185 (71.2)	

CAD, Coronary artery disease; n, number of participants; and SNP, single-nucleotide polymorphism.

The *P*-value of the genotypes was calculated by Fisher’s exact test; ^*^P < 0.05.

**Table 4:** Genotype and allele distributions in Uygur patients with CAD and control participants

		Total			Men			Women		
		CAD *n* (%)	Controls *n* (%)	*P*	CAD *n* (%)	Controls *n* (%)	*P*	CAD *n* (%)	Controls *n* (%)	*P*
SNP1 rs2236722 genotype	AA	399 (99)	370 (95.6)	0.03	314 (99.4)	163 (97)	0.04	85 (97.7)	207 (96.7)	0.653
	GA	4 (1)	17 (4.4)		2 (0.6)	5 (3)		2 (2.3)	7 (3.3)	
										
SNP2 rs2304463 genotype	CC	108 (26.8)	87 (22.5)	0.365	86 (27.2)	26 (15.5)	0.009	22 (25.3)	61 (27.9)	0.589
	CA	191 (47.4)	192 (49.6)		152 (48.1)	87 (51.8)		39 (44.8)	105 (47.9)	
	AA	104 (25.8)	108 (27.9)		78 (24.7)	55 (32.7)		26 (29.9)	53 (24.2)	
										
Dominant model	CC	108 (26.8)	87 (22.5)	0.159	86 (27.2)	26 (15.5)	0.004	22 (25.3)	61 (27.9)	0.649
	CA + AA	295 (73.2)	300 (73.5)		230 (72.8)	142 (84.5)		65 (74.7)	158 (72.1)	
Recessive model	AA	104 (25.8)	108 (27.9)	0.505	78 (24.7)	55 (32.7)	0.059	26 (29.9)	53 (24.2)	0.305
	CC + CA	299 (74.2)	279 (22.1)		238 (75.3)	113 (67.3)		61 (70.1)	166 (75.8)	
Additive model	CA	191 (47.4)	192 (49.6)	0.533	152 (48.1)	87 (51.8)	0.44	39 (44.8)	105 (47.9)	0.622
	CC + AA	212 (52.6)	195 (50.4)		164 (51.9)	81 (48.2)		48 (55.2)	114 (52.1)	
										
SNP3 rs4646 genotype	CC	172 (42.7)	210 (54.3)	0.001	140 (44.3)	90 (53.6)	0.007	32 (36.8)	120 (54.8)	0.017
	CA	185 (45.9)	129 (33.3)		143 (45.3)	52 (31)		42 (48.3)	77 (35.2)	
	AA	46 (11.4)	48 (12.4)		33 (10.4)	26 (15.5)		13 (14.9)	22 (10)	
										
Dominant model	CC	172 (42.7)	210 (54.3)	0.000	140 (44.3)	90 (53.6)	0.002	32 (36.8)	120 (54.8)	0.034
	CA + AA	231 (57.3)	177 (45.7)		176 (55.7)	78 (46.4)		55 (63.2)	99 (45.2)	
Recessive model	AA	46 (11.4)	48 (12.4)	0.668	33 (10.4)	26 (15.5)	0.107	13 (14.9)	22 (10)	0.225
	CC + CA	357 (88.6)	339 (87.6)		283 (89.6)	142 (84.5)		74 (85.1)	197 (90)	
Additive model	CA	185 (45.9)	129 (33.3)	0.001	143 (45.3)	52 (31)	0.052	42 (48.3)	77 (35.2)	0.004
	CC + AA	218 (54.1)	238 (66.7)		173 (54.7)	116 (69)		45 (51.7)	142 (64.8)	
										
SNP4 rs4275794 genotype	TT	278 (69)	263 (68)	0.953	214 (67.7)	105 (62.5)	0.48	64 (73.6)	158 (72.1)	0.503
	CT	109 (27)	108 (27.9)		87 (27.5)	55 (32.7)		22 (25.3)	53 (24.2)	
	CC	16 (4)	16 (4.1)		15 (4.7)	8 (4.8)		1 (1.1)	8 (3.7)	
										
Dominant model	TT	278 (69)	263 (68)	0.757	214 (67.7)	105 (62.5)	0.249	64 (73.6)	158 (72.1)	0.802
	CT + CC	125 (31)	124 (32)		102 (32.3)	63 (37.5)		23 (26.4)	61 (27.9)	
Recessive model	CC	16 (4)	16 (4.1)	0.907	15 (4.7)	8 (4.8)	0.994	1 (1.1)	8 (3.7)	0.242
	TT + CT	387 (96)	371 (95.9)		301 (95.3)	160 (95.2)		86 (98.9)	211 (96.3)	
Additive model	CT	109 (27)	108 (27.9)	0.787	87 (27.5)	55 (32.7)	0.231	22 (25.3)	53 (24.2)	0.842
	TT + CC	294 (73)	279 (72.1)		229 (72.5)	113 (67.3)		65 (74.7)	166 (75.8)	

CAD, Coronary artery disease; n, number of participants; and SNP, single-nucleotide polymorphism.

The *P*-value of the genotypes was calculated by Fisher’s exact test; *P* < 0.05.

Table 3 shows that among the total Han population and male Han subjects, all SNP genotypes were not significantly different between CAD patients and control subjects (*P >* 0.05) in the dominant, recessive, and additive models. However, for female subjects, the SNP1 (rs2236722) and SNP3 (rs4646) genotypes in the dominant model (CC vs. CA + AA) were significant different between the CAD patients and control subjects (*P =* 0.01 and *P =* 0.05, respectively).

Table 4 shows that in the Uygur population, two loci, SNP1 (rs2236722) and SNP3 (rs4646), were significantly associated with CAD. The distribution of the SNP1 genotypes was significantly different between CAD and control subjects (total: *P =* 0.003, men: *P =* 0.04). Additionally, the distribution of the rs4646 genotypes in the dominant model (CC vs. CA + AA) and additive model (CA vs. CC + AA) was significantly different between CAD and control subjects (*P =* 0.001, *P =* 0.000, and *P =* 0.001, respectively). Similar to the men, the differences among women remained statistically significant. However, there was no significant difference between CAD and control subjects regarding the recessive model (AA vs. CC + CA) (*P =* 0.668). Among men, the distribution of the SNP2 genotypes in the dominant model (CA + AA vs. CC) was significantly different between the CAD and control subjects (*P =* 0.009 and *P =* 0.004, respectively). However, the SNP4 genotypes were not significantly different between CAD patients and control subjects (both *P >* 0.05) in the dominant, recessive, and additive models.

### Logistic regression analyses

Associations of the CYP19A1 rs2236722 and rs10046 polymorphisms with the traditional risk factors of CAD were observed. In the Uygur population, a multivariable logistic regression analysis was performed, combining the genotypes with the following variables: age, gender, hypertension, diabetes, smoking, drinking, BMI, TG, and LDL-C. After multivariate adjustment, SNP1 (rs2236722) remained significantly associated with CAD in terms of genotype (OR = 0.271, 95% CI: 0.077–0.953, *P =* 0.042). After multivariate adjustment, SNP3 (rs4646) remained significantly associated with CAD in the dominant model (CC vs. CA + AA) (total: OR = 0.483, 95% CI: 0.338–0.690, *P =* 0.000; and men: OR = 0.455, 95% CI: 0.293–0.704, *P =* 0.000) and the additive model (CA vs. CC + AA) (total: OR = 1.844, 95% CI: 1.300–2.617, *P =* 0.001; men: OR = 1.651, 95% CI: 1.087–2.507, *P =* 0.019; and women: OR = 2.179, 95% CI: 1.103–4.305, *P =* 0.025), as shown in Table 5. In the Han population, because there was no difference in genotype distribution, further multivariable logistic regression analyses were not performed.

**Table 5:** Multiple logistic regression analysis for CAD patients and control subjects of the Uygur Chinese population

	Total		Men		Women	
	OR (95% CI)	*P*	OR (95% CI)	*P*	OR (95% CI)	*P*
rs2236722 (SNP1) genotype	0.271 (0.077–0.953)	0.042	0.177 (0.03–1.046)	0.056	0.295 (0.032–2.706)	0.280
rs4646 (SNP3) Dominant model (CC vs. CA + AA)	0.483 (0.338–0.690)	0.000	0.455 (0.293–0.704)	0.000	0.585 (0.301–1.138)	0.114
rs4646 (SNP3) Additive model (CA vs. CC + AA)	1.844 (1.300–2.617)	0.001	1.651 (1.087–2.507)	0.019	2.179 (1.103–4.305)	0.025

OR, odds ratios; and 95% CI, 95% confidence intervals; *P* < 0.05.

### SNPs and circulating sex hormone levels

We analyzed the association of four SNPs in the CYP19A1 gene with circulating hormone and aromatase levels in normal postmenopausal women. Statistically significant associations with hormone levels were observed in the two ethnic groups.

Supplementary Table 1 shows that in the Uygur CAD population, SNP3(rs4646) is significantly associated with differences in estradiol, testosterone, and aromatase levels (*P =* 0.040, *P =* 0.007, and *P =* 0.009, respectively), and this association remained significant after adjustments for age, BMI, BUN, and glucose levels (*P =* 0.05, *P =* 0.03, and *P =* 0.03, respectively). In the Uygur control population, SNP3 was significantly associated with differences in all circulating hormone and aromatase levels (*P =* 0.000 and *P =* 0.007, respectively), and the association remained significant after adjustments for age, BMI, BUN, and glucose levels (*P =* 0.000 and *P =* 0.026, respectively). In the Han CAD population (data not shown), rs4646 was associated with differences in testosterone and aromatase levels (*P =* 0.003 and *P =* 0.007, respectively). However, the association was not statistically significant after adjustment. In the control Han population, this SNP at rs4646 was associated only with differences in estradiol levels (*P =* 0.009), and the adjusted model remained significant (*P =* 0.034). Several loci were not related to hormone levels and CAD (both *P* > 0.05).

Table 6 shows the results of a multivariable logistic regression analysis combining hormone levels and aromatase levels with the following variables: rs4646 (CC); the incidence of diabetes and hypertension; age; nation(Han and Uygur); and BMI, which are major confounding factors of CAD. After multivariate adjustment, CAD remained significantly associated with estradiol (OR = 0.889, 95% CI:0.817–0.969, *P =* 0.007) and aromatase (OR = 0.947, 95% CI:0.936–0.957, *P =* 0.000) but not testosterone (OR = 6.894, 95% CI:0.876–4.967, *P =* 0.067).

**Table 6:** Multiple logistic regression analysis of the association between female sex hormone and aromatase levels among normal Chinese postmenopausal female CAD patients and control subjects

Factor	OR (95% CI)	*P*
Estradiol (pg/ml)	0.889 (0.817–0.969)	0.007
Testosterone	6.894 (0.876–4.967)	0.067
Aromatase (IU/L)	0.947 (0.936–0.957)	0.000
rs4646 (CC)	0.493 (0.261–0.93)	0.029
Age	1.047 (1.005–1.091)	0.027
nation	0.914 (0.428–1.953)	0.816
BMI	0.98 (0.912–1.052)	0.577
EH	1.229 (0.665–2.271)	0.511
Diabetes	3.253 (1.409–7.511)	0.006

OR, odds ratios; and 95% CI, 95% confidence intervals; *P* < 0.05.

**Figure 2:**
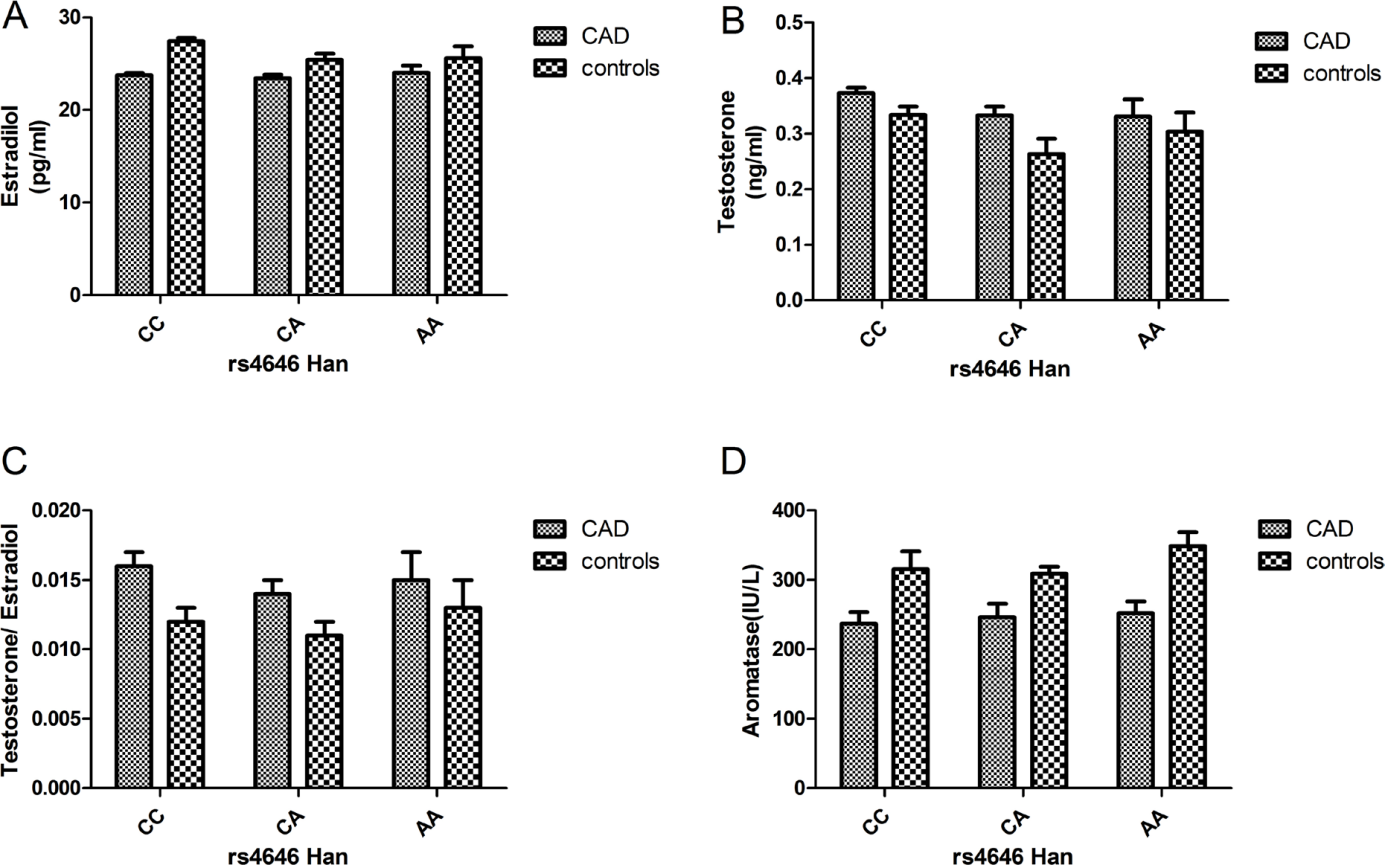
**(A–D)** Circulating sex hormone and aromatase levels (after adjustment; mean ± SE) in Han postmenopausal female CAD and control subjects with each rs4646 genotype (CAD: CC: 111, CA: 47, and AA: 12; controls: CC: 50, CA: 15, and AA: 4).

**Figure 3:**
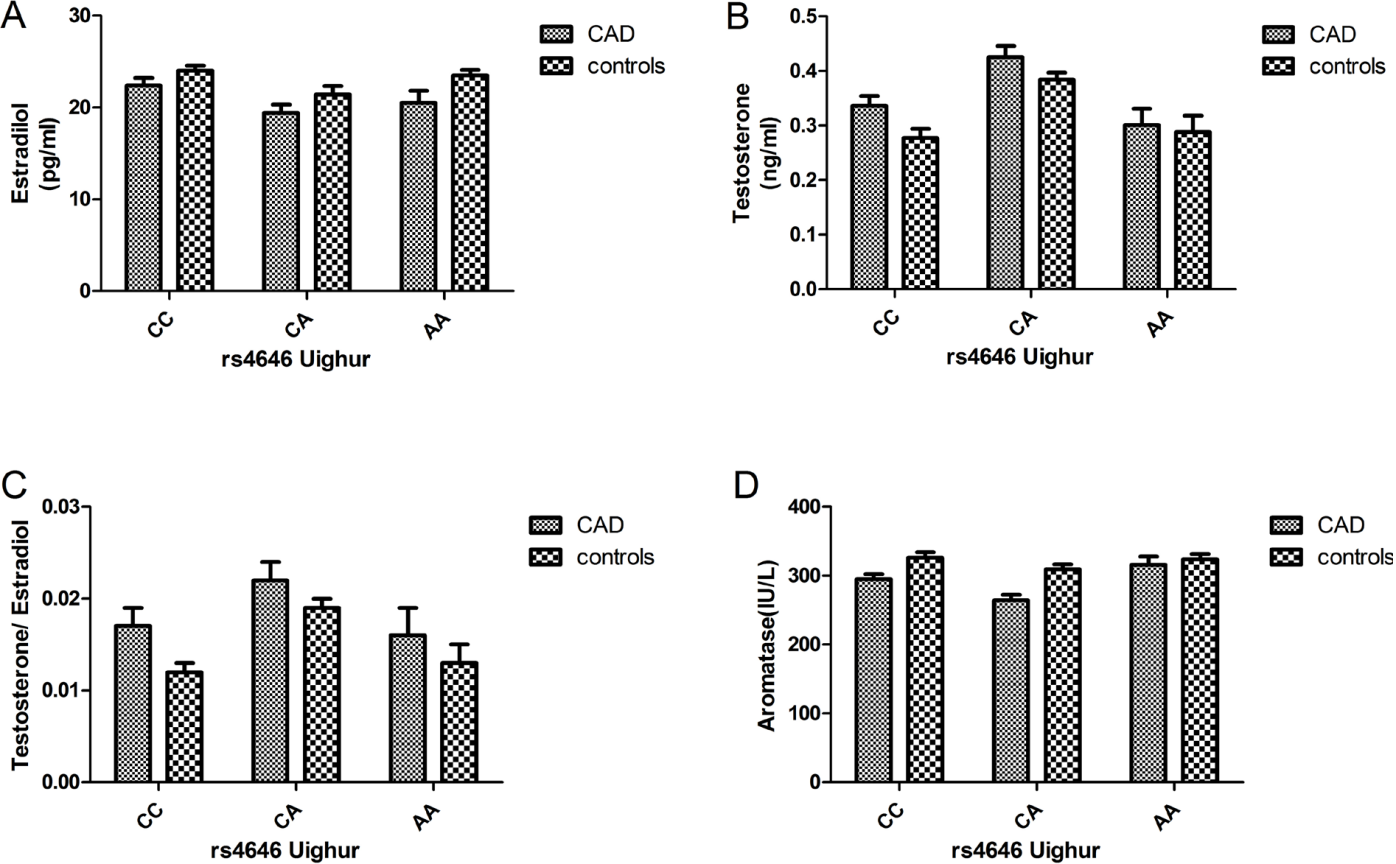
**(A–D)** Circulating sex hormone and aromatase levels (after adjustment; mean ± SE) in Uygur postmenopausal female CAD and control subjects with each rs4646 genotype (CAD: CC: 32, CA: 26, and AA: 12; controls: CC: 47, CA: 76, and AA: 14).

In both the Uygur and Han populations, estrogen levels were higher in the control group than in the case group. However, the testosterone levels were higher in the case group than in the control group, and the same phenomenon was observed for testosterone/estrogen levels. With respect to aromatase levels, the control group showed higher levels than the case group. Overall, the hormone levels in the Uygur population were lower than those in the Han population (Figures 2 and 3).

## DISCUSSION

### Findings

In the present study, we observed that variations in the CYP19A1 gene were associated with CAD in a Uygur population in China. We further examined the potential associations between genotypes and the levels of sex hormones in postmenopausal Chinese women. The data indicated that SNP3 (rs4646) was strongly associated with estradiol, testosterone, and aromatase levels.

The human CYP19A1 gene is located on chromosome 15q21.2 and encodes an ∼130 kb sequence that includes nine (II-X) coding exons [9]. In postmenopausal women, a critical step in estrogen biosynthesis involves the formation of C18 estrogens (estrone and estradiol) from C19 androgens (androstenedione and testosterone). The aromatase gene CYP19A1 is critical for this transformation [10–11]. Previous studies have mainly focused on the relationship between polymorphisms in the CYP19A1 gene and breast cancer [12–15]. CYP19A1 is related to estrogen, and both animal experiments and clinical observations have demonstrated that the occurrence of CAD is related to the levels of sex hormones in the body [16–18]. Thus, estrogen likely plays a protective role in the cardiovascular system; therefore, it is not surprising that CYP19A1 polymorphisms are associated with CAD.

In this study, we recruited subjects from the ethnic minorities in Xinjiang, China, including Uygur and Han populations. We observed that polymorphisms in CYP19A1 were associated with CAD in the Uygur population. In the total and male populations, the frequency of GA genotypes for SNP1 (rs2236722) was higher in control subjects than in CAD subjects, and there were significant differences in the genotypes after multivariate adjustments for the confounding factors of CAD, indicating that the GA genotype may be protective against CAD. Notably, SNP1 (rs2236722) is a missense mutation observed at 39 loci, in which Trp is changed to Arg in exon 2. The study of CYP19A1 gene function is ongoing. Using the SNP1 mutation, we have constructed a mutant plasmid and a wild-type plasmid, and cell and animal experiments continue to be carried out simultaneously; however, detailed results are not yet available. Nevertheless, these studies will greatly contribute to a better understanding of the CYP19A1 gene.

Because CYP19A1 directly affects estrogen levels, many scholars believe that polymorphisms of the CYP19A1 gene may affect the level of sex hormones in different individuals. Christopher A. Haiman [19] found that 103 polymorphisms of CYP19 were in linkage disequilibrium European white women, and haplotype construction analysis showed that the CYP19 gene-encoding region close to the 5′ non-encoding region was associated with significantly increased postmenopausal estrogen levels. In particular, the greater than 67 kb region, including the coding region, 3′UTR, and tissue-specific promoters I.2, I.6, I.3 and PII, is associated with the conversion of androgens to estrogens. The Chinese scholar Hui Cai [20] studied postmenopausal women in China and found that an AA genotype near the I.4 promoter site in rs1902584 was associated with low levels of estradiol in overweight and postmenopausal women. Additionally, the Japanese scholar Kumiko Kidokoro [21] identified 25 SNP loci in the CYP19 gene among 100 postmenopausal Japanese women and found that rs12148604 in the 3′UTR was weakly associated with estrone and testosterone levels.

In our research, we found that the frequency of the CA genotype for SNP3 (rs4646) was higher in CAD subjects than in control subjects and that there were significant differences among the genotypes in the additive models in the Uygur population. Moreover, this difference remained significant after multivariate adjustments for the confounding factors of CAD, indicating that the CA genotype may be a risk factor for CAD. In contrast, the frequency of the CC genotype was higher in control subjects than in CAD subjects, and there were significant differences among the genotypes in the dominant models. After multivariate adjustments for the confounding factors of CAD, the difference remained significant, indicating that the CC genotype may be protective against CAD.

The results showed that sex hormones levels were significantly affected by SNP3 (rs4646) in the 3′untranslated region (UTR). In CAD and control groups, the protective CC genotype of rs4646 was associated with higher estradiol and aromatase levels than the other two homozygous genotypes, but the testosterone level was lower in individuals with the CC genotype than the other two homozygous genotypes. There were also significant differences among different races; for example, the levels of estradiol and aromatase were higher among Uygur normal postmenopausal women than Han normal postmenopausal women. We further conducted multivariable logistic regression analyses combining hormone and aromatase levels with the rs4646 CC genotype and the major confounding factors of CAD. After multivariate adjustment, CAD remained significantly associated with estradiol (OR = 0.889, 95% CI:0.817–0.969, *P =* 0.007) but not testosterone (OR = 6.894, 95% CI:0.876–4.967, *P =* 0.067). CAD also remained significantly associated with aromatase after adjustments for confounders of CAD (OR = 0.947, 95% CI:0.936–0.957, *P =* 0.000).

Our findings are consistent with those of previous studies, showing that there is an association between polymorphisms in the 3′ UTR (rs10046) and sex hormone levels [22–23]. Notably, previous studies have also identified SNPs in the same 3′ UTR. The HapMap database showed rs10046 in four ethnicities: JPT (Japanese individuals from Tokyo), HCB (Han Chinese individuals from Beijing), CEU (American individuals from Utah with northern and western European ancestry), and YRI (Yoruba individuals from Ibadan and Nigeria) [19–24]. Therefore, the results of the present study for SNP3 combined with those of other studies suggest that there is a functional region in the 3′ UTR of CYP19A1 and imply that its association with estradiol levels may reflect the presence of another polymorphism in the noncoding region that is in linkage disequilibrium with the SNPs in the 3′ UTR and influence hormone levels among postmenopausal women of various ethnic backgrounds (two ethnic groups were included in the present study). At the same time, these SNPs may play a role in the occurrence and development of CAD. Therefore, a better understanding of the pathogenesis of coronary heart disease may help improve prognosis.

We found that aromatase and estrone levels are reduced and that testosterone levels are increased in postmenopausal women with CAD. This finding may indicate that in postmenopausal women, aromatase activity decreases with increasing testosterone levels and estrone levels are decreased compared with controls. Moreover, the appropriate proportion of testosterone/estrogen levels protects women, but when the levels are imbalanced, there may be a harmful effect [25]. Polycystic ovary syndrome (PCOS) has been hypothesized to be caused by functional ovarian hyperandrogenism (FOH) resulting from dysregulated androgen secretion [26]. Indeed, women with multiple ovarian syndrome are at high risk for coronary heart disease [27]. Aromatase affects androgen levels in women after menopause because aromatase converts testosterone to estradiol. Studies have shown that the inhibition of aromatase activity can reduce the levels of estrogen, resulting in excessive accumulation of the androgen precursor [28]. In postmenopausal women, the prevalence of coronary heart disease is increased; however, whether increased androgen levels, gender imbalance, or decreased aromatase activity contribute to this effect requires further investigation. An increasing number of studies have tested the effects of genetic variations in the aromatase (CYP19A1) gene on CAD; however, whether the CYP19A1 gene is associated with CAD remains controversial.

Although the nature of our study is similar to previously published studies on CAD in other Han Chinese populations, this is the first study to investigate and compare CYP19A1 gene polymorphisms, sex hormones levels, and potential risk factors of CAD in different ethnicities in Xinjiang, northwestern part of China with a unique lifestyle and natural environment. Xinjiang is part of the ancient Silk Road and borders eight countries. There are more than 13 ethnic groups living in this area. The Uygur people account for 46% of the population, and the Han people account for 40%. There is an important difference between the habits and diets of the two ethnic groups. In addition, the Uygur are an admixed population, originating from intermarriages between Caucasians and East Asians, thus contributing another important difference between the Uygur and Han populations.

### Limitations and shortcomings

There are several limitations in the present study. First, the source of CAD patients was limited to the First Affiliate Hospital of Xinjiang Medical University, and these subjects may possess some risk factors of cardiovascular disease. Second, the relatively small sample size of this study may have contributed to weak statistical significance and wide CIs in the estimation of the OR. Third, there is a lack of individual genetic background information for the Uygur subjects. Finally, although we confirmed that CYP19A1 was associated with CAD and the levels of sex hormones and aromatase in postmenopausal Chinese women, we did not assess the polymorphic loci. Moreover, the present study lacked functional validation. However, assessments of the functional outcomes and correlations with hormonal levels are currently ongoing. Nevertheless, a prospective cohort study with a reasonably long study duration is required to obtain evidence with higher quality and reliability.

## MATERIALS AND METHODS

### Ethics approval of the study protocol

This study was approved by the Ethics Committee of the First Affiliated Hospital of Xinjiang Medical University (Xinjiang, China) and conducted according to the standards of the Declaration of Helsinki. Written informed consent was obtained from each participant, including explicit permission for DNA analysis and the collection of relevant clinical data.

### Study population

We randomly recruited 611 Han (421 men and 190 women) and 403 Uygur (316 men and 87 women) patients with CAD as well as 453 Han and 387 Uygur individuals as ethnically and geographically matched control subjects. Among these individuals, 498 postmenopausal women (265 Han and 233 Uygur individuals) were selected from a subset of participants older than 55 years old who had not menstruated for 1 year or more and had not received hormone replacement therapy for at least 3 months prior to sampling. All subjects were inpatients of the First Affiliated Hospital of Xinjiang Medical University from 2013 to 2016. CAD was defined as the presence of at least one major coronary artery with stenosis affecting more than 50% of the luminal diameter on coronary angiography. Control subjects also underwent a coronary angiogram and were confirmed to be free of coronary artery stenosis; they also did not exhibit clinical or electrocardiographic evidence of myocardial infarction (MI) or CAD. However, some control subjects had cardiovascular risk factors, such as essential hypertension (EH), diabetes mellitus (DM), or hyperlipidemia, suggesting that the control group was exposed to the same risk factors of CAD, but the results of the coronary angiogram were normal. All information and data (including EH, DM, hyperlipidemia, and smoking) were collected from all study subjects, and these individuals were matched between two CAD and control cohorts. Patients were diagnosed with hypertension if they were on antihypertensive medication or if their systolic blood pressure (SBP) was > 140 mm Hg or diastolic blood pressure (DBP) was > 90 mm Hg in 3 measurements. DM was diagnosed according to the criteria of the American Diabetes Association [29]. Smoking status was dichotomized as smokers (current and ex-smokers) or non-smokers. Additionally, all participants with impaired renal function, malignancies, connective tissue disease, chronic inflammatory disease, multiple organ failure, or mental disorders were excluded from the present study. All study participants provided written informed consent.

### Sex hormone analysis in normal postmenopausal women

Sex hormone measurements were obtained using stored serum samples from the 498 postmenopausal women. After conducting comparative studies, which established that there were only minor differences between values obtained from the plasma and serum samples from the same individual, the remaining measurements were obtained from plasma samples. Sex hormone analyses were sequentially performed for estradiol and then testosterone using the available plasma or serum from each subject; 100% of the subjects had sufficient plasma or serum to complete the estradiol and testosterone measurements. Total testosterone and estradiol was measured using a radioimmunoassay kit (T*KIT-B10PZA and E2*KIT-B05PZA, Beijing North Institute of Biological Technology, Beijing, China) based on GC-2016γ using a radioimmunoassay counter at the Clinical Nuclear Medicine Department of the First Affiliated Hospital of Xinjiang Medical University. Total testosterone and estradiol precision was determined based on batch variation of cv < 10%, inter-batch variation of cv < 15%, a concentration of 3.1 nmol/L, and a sensitivity limit of 0.14 nmol/L. Plasma P450 aromatase levels were measured using an enzyme-linked immunosorbent assay (Human Aromatase ELISA Kit, Beijing Kamal Shu Biotechnology Institute, Beijing, China). Absorbance (OD) was measured at 450 nm using a BIO-RAD xMark^™^ Microplate Spectrophotometer, and a standard curve was used to calculate the human aromatase concentration based on a batch variation of cv < 9% and an inter-batch variation of cv < 11%.

### Biochemical analyses

For the biochemical analyses, 5 ml of fasting venous blood was drawn by venipuncture from all participants. The blood samples were collected and centrifuged at 4,000 × g for 5 min to separate the plasma from the blood cells. Genomic DNA was extracted using the standard phenol-chloroform method. The DNA samples were stored at –80°C for future analysis. For the analyses, the DNA was diluted to a 50-ng/µl analysis. Fo. The plasma concentrations of glucose, total cholesterol (TC), triglyceride (TG), high-density lipoprotein cholesterol (HDL-C), low-density lipoprotein cholesterol (LDL-C), blood urea nitrogen (BUN), creatinine (Cr), and uric acid (UA) were measured using standard methods at the Central Laboratory of the First Affiliated Hospital of Xinjiang Medical University as previously described [30–31].

### Genotyping the CYP19A1 gene

**Figure 4:**
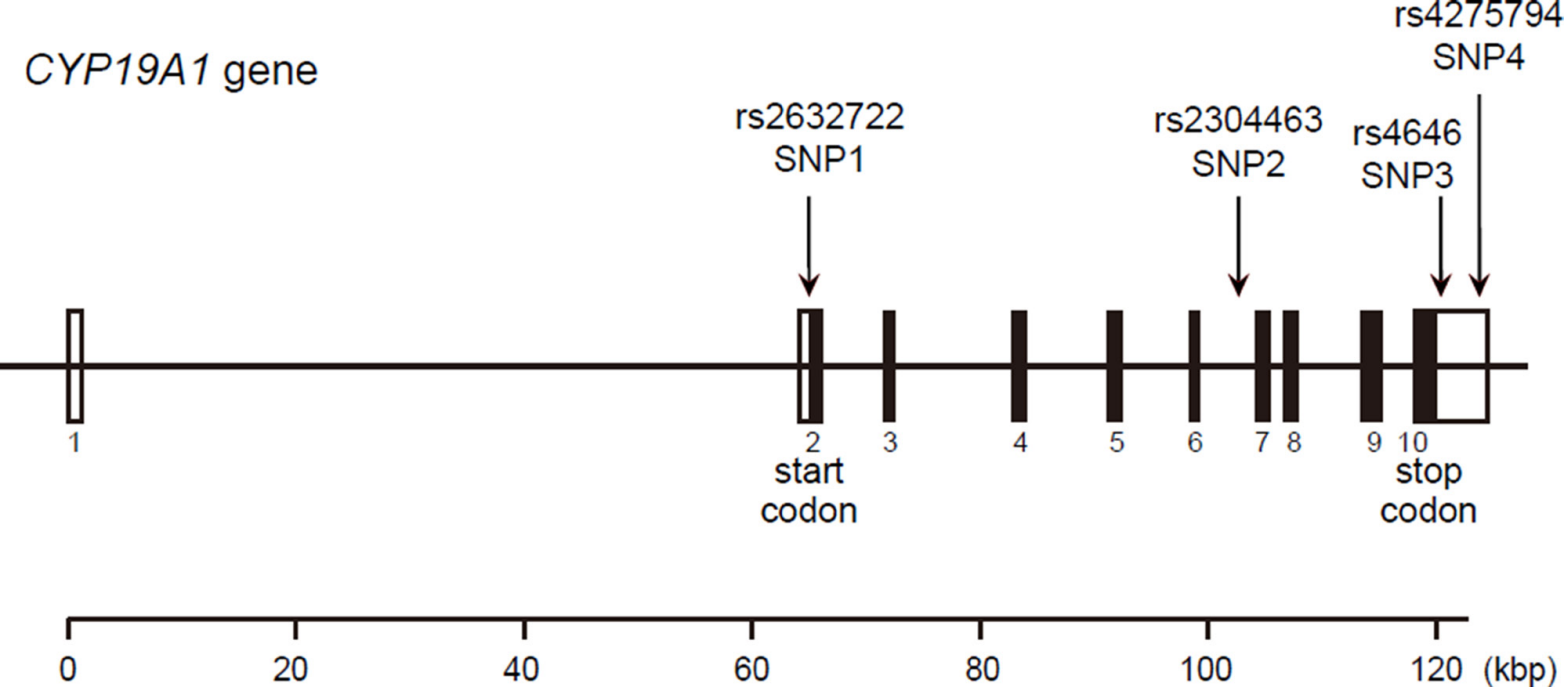
Structure of the human CYP19A1 gene Boxes indicate exons, and lines indicate introns and intergenic regions. Filled boxes indicate coding regions. Arrows mark the locations of polymorphisms.

We randomly selected 48 DNA samples with which to screen CYP19A1 gene mutation sequences. Mutation screening of 11 exons of the CYP19A1 gene was conducted at Genesky Biotechnologies Inc. (Shanghai, China). A total of 29 SNP loci were identified. After extensive review of the literature and information in the National Center for Biotechnology Information SNP database (http://www.ncbi.nlm.nih.gov/ SNP) and HuGE Navigator database (http://hugenavigator.net/), Haploview 4.2 software and the HapMap phase II database were used to obtain four tagged SNPs: rs2236722 (EXON2, missense, c.115T > C, p.Trp39Arg); rs2304463 (INTRON6, c.744–106T > G); rs4646 (3′ UTR, c.*161T > G); and rs4275794 (3′ UTR, c.*1888A > G) for the Chinese population based on a minor allele frequency (MAF) ≥ 0.05 and linkage disequilibrium patterns with r ^2^ ≥ 0.8 as a cutoff. We designated these SNPs as SNP1, SNP2, SNP3 and SNP4 in order of increasing distance from the 5′ end of the gene (Figure 4).

SNP genotyping was performed using the improved multiplex ligation detection reaction (iMLDR) technique (Genesky Biotechnologies Inc., Shanghai, China) as described by Dengming He et al. [32]. Randomly selected DNA samples from each genotype were sequenced to validate the genotyping using the ligation detection reaction method. The results of the ligation detection reaction method were consistent with the results of sequencing.

### Statistical analysis

Statistical analyses were performed using SPSS 22.0 for Windows (SPSS Institute, Chicago, USA). Statistical significance was indicated by a *P*-value < 0.05. All continuous variables (e.g., age, TC, TG, HDL-C, LDL-C, and body mass index (BMI)) are presented as the mean ± standard deviation (SD), and the differences between the CAD and control groups were analyzed using independent-sample *t*-tests. All the variables (e.g., the frequencies of smoking, drinking, DM, and CYP19A1 genotypes) and the Hardy-Weinberg equilibrium were analyzed using the χ^2^ test or Fisher’s exact test, as appropriate. Logistic regression analyses with effect ratios (odds ratio [OR] and 95% confidence interval (CI)) were used to assess the contributions of the major risk factors. A general linear model (GLM) analysis was used to test for associations between SNP genotypes and sex hormone and aromatase levels after adjusting for confounding variables.

A two-degree-of-freedom likelihood ratio test for homogeneity of the effect of each SNP was performed. A *P*-value ≤ 0.05 was considered statistically significant, and a value between 0.05 and 0.1 was considered marginally significant.

## CONCLUSIONS

In summary, the results of the present study provide additional information concerning CYP19A1 polymorphisms and their associations with CAD, and to our knowledge, this study is the first to examine the association between tag SNPs in CYP19A1 and sex hormone levels in postmenopausal Xinjiang Chinese women. The results showed an association between estrone, testosterone, and aromatase levels in the SNP3 (rs4646) region in the 3′ UTR of CYP19A1. This association may represent a potential susceptibility marker of hormone-dependent diseases in postmenopausal women. Additional studies and functional validation of these associations are needed to develop better hormone replacement therapies for the prevention and treatment of cardiovascular disease.

## SUPPLEMENTARY MATERIALS TABLE





## Abbreviations

CAD: Coronary artery disease
SNPs: Single-nucleotide polymorphisms
E2: 17β-estradiol
T: Testosterone
A: Aromatase
