# Supplementary material for: CYP19A1 polymorphisms associated with coronary artery disease and circulating sex hormone levels in a Chinese population
Source: Oncotarget. 2017 Oct 7;8(57):97101–13. doi: 10.18632/oncotarget.21626 (PMC5722548; doi:10.18632/oncotarget.21626)
Supplement: Supplementary file 1 [file oncotarget-08-97101-s001.pdf]

CYP19A1 polymorphisms associated with coronary artery disease and circulating sex hormone levels in a Chinese population

SUPPLEMENTARY MATERIALS

Supplementary Table 1: Mean circulating hormone levels (ratios) and aromatase levels by CYP19A1 genotype in normal Uygur postmenopausal women. See Supplementary_Table_1
